# Three-dimensional SEM, TEM, and STEM for analysis of large-scale biological systems

**DOI:** 10.1007/s00418-022-02117-w

**Published:** 2022-07-12

**Authors:** Snježana Radulović, Sowmya Sunkara, Reinhard Rachel, Gerd Leitinger

**Affiliations:** 1 Division of Cell Biology, Histology and Embryology, Research Unit Electron Microscopic Techniques, Gottfried Schatz Research Center, Medical University of Graz, 8010 Graz, Austria; 2 Centre for Electron Microscopy, University of Regensburg, 93053 Regensburg, Germany

**Keywords:** Electron tomography, Large volumes, Structural cell biology, 3D reconstruction, Correlative light and electron microscopy, Array tomography

## Abstract

A major aim in structural cell biology is to analyze intact cells in three dimensions, visualize subcellular structures, and even localize proteins at the best possible resolution in three dimensions. Though recently developed electron microscopy tools such as electron tomography, or three-dimensional (3D) scanning electron microscopy, offer great resolution in three dimensions, the challenge is that, the better the resolution, usually the smaller the volume under investigation. Several different approaches to overcome this challenge were presented at the Microscopy Conference in Vienna in 2021. These tools include array tomography, batch tomography, or scanning transmission electron tomography, all of which can nowadays be extended toward correlative light and electron tomography, with greatly increased 3D information. Here, we review these tools, describe the underlying procedures, and discuss their advantages and limits.

## Introduction

A major requirement of structural cell biology is that the organelles under investigation need to be visualized in three dimensions to gain full insight into structure–function relationships. Many structures would be misunderstood if relying solely on single sections. In a coarse approximation, a single section contains—at best—about 1% of the information from the cellular object under investigation, and misunderstandings increase, the more complex the structures. To satisfy the need for 3D analysis, a range of methods that enable three-dimensional analysis using either scanning or transmission electron microscopes have become available. However, high-resolution in three dimensions comes at the cost of severe limits in the volume that can be examined. An electron tomogram, for example, though providing 3D resolution at the nanometer scale, is made of structures within sections that are only 200–400 nm thick. Thus, a major challenge in structural biology is to describe the 3D structure of large volumes, such as analyzing a given intact cell in 3D in its integrity.

Several recent, new developments have addressed this problem and allow the investigation of larger volumes in 3D. These include transmission electron microscopy (TEM), scanning electron microscopy (SEM), and scanning transmission electron microscopy (STEM). In many approaches, light microscopy is now included in the workflows and combined with electron microscopy, resulting in so-called correlative light and electron microscopy (CLEM) procedures.

Here, we review such solutions that were presented in the “3D S(T)EM for analysis of large-scale biological systems” session at the Microscopy Conference in 2021 (chairs: G. Leitinger and R. Rachel) (Bernardi et al. 2021). Depending on the scientific questions addressed, the solutions range from CLEM, including fluorescence or even superresolution light microscopy, to 3D scanning electron microscopy methods that allow an astonishingly large sample volume, to ways of increasing the section thickness in transmission electron microscopy (Britz et al. 2021a; Cserép and Dénes 2021; Bekel et al. 2021; Botzenhart et al. 2021; Rachel et al. 2021a; Yamaguchi et al. 2021).

We first provide an overview of current cutting-edge methods for analyzing samples in 3D with electron microscopes, and then describe the selected solutions as presented by six different groups at this conference, and discuss their possible drawbacks and major advantages. It is not our aim to reiterate the content of other, already published reviews; for this, the reader is rather referred to review articles published elsewhere (Muller-Reichert 2021). Instead, we aim to explain novel solutions and cite key literature in close connection to the conference presentations.

## Methods

As will be shown in the chapter “Solving scientific questions with 3D information from large volumes”, the solutions presented for obtaining 3D information from large-scale samples are manifold and often based on combinations of different techniques. Here, we provide a brief overview of those techniques.

### CLEM

Correlative light and electron microscopy (CLEM) is a powerful technique that combines any type of optical (most frequently, fluorescent) and (various means of) electron microscopy. This approach enables the correlation of two types of information: cell function deduced from the subcellular location of the fluorescently labeled macromolecule and the ultrastructure of the same region of interest of the same sample. CLEM can be performed using separate instruments, even in separate facilities. However, an integrated version offers simultaneous imaging on a device that offers both fluorescent and SEM capabilities (Liv et al. 2013).

### Three-dimensional SEM

The traditional approach for obtaining three-dimensional information at high resolution is serial section transmission electron microscopy (ssTEM). This is done with conventional equipment: an ultramicrotome to produce serial sections that are placed on slot grids supported by a polymer film, and a transmission electron microscope. One splendid proof of the usefulness of this technique is the description of the structure of the nervous system of *Caenorhabditis elegans*, including the connectivity between the 302 neurons involved (White et al. 1986). However, hand-producing serial sections requires considerable skill and time and carries the risk of losing sections. Thus, newer methods that make use of the characteristics of backscattered electron (BSE) detectors in scanning electron microscopes have been developed. The denser and heavier the atoms underneath the surface of the sample, the higher the signal. When sections of resin-embedded tissue are stained with heavy metals and visualized with a BSE detector, the resulting contrast can be inverted. After inversion, the images look similar to conventional TEM micrographs. Thus, such images can be readily interpreted by experienced electron microscopists. Several different, 3D scanning electron microscopy methods make use of BSE detectors.

### Array tomography

Serial sectioning was first described in the 1950s by Hillier and Gay (Hillier and Gettner 1950; Gay and Anderson 1954). Over the years, numerous improvements have been made to the method. Based on this experience, array tomography (AT) was established and described by Micheva and Smith (2007). This powerful method is used for the 3D visualization of large volumes of samples. Currently, there are many variations, but the procedure always requires fixing and embedding the sample in resin. The block is then ultrathin-sectioned, resulting in a collection of ordered arrays of serial sections on solid substrates.

The variants of array tomography are:

1. Heavy-metal staining and direct SEM imaging. A useful tool for this is a well-developed ultramicrotome called automated tape collection ultra microtome (ATUMTome) (Schalek et al. 2011). This special microtome allows the placement of a series of ultrathin sections on a plastic tape and their visualization using a scanning electron microscope. The tape is made of Kapton, which is opaque but enables a nearly unlimited number of sections to be collected, visualized, and preserved. There is an additional option of immunogold labeling.
2. Fluorescent labeling with possible elution and relabeling and fluorescence microscopy, which may be followed by heavy-metal staining and SEM imaging (correlative array tomography, CAT) (Oberti et al. 2011). Here, a limited number of sections are collected on glass coverslips or other transparent material (Wacker and Schroeder 2013).

Another approach similar to AT is to have multiple serial sections arranged on glass coverslips followed by antibody labeling and superresolution LM analysis. One such example is *tomo*STORM, a method developed by Nanguneri et al. (2012), in which consecutive sections of structurally intact brain tissue are organized on a ribbon labeled with photoswitchable organic fluorophores and serially imaged by stochastic optical reconstruction microscopy (STORM).

Array tomography combines the advantages of fluorescent microscopy and SEM. It allows high-resolution imaging of large fields and big volumes, combined with the usage of detecting numerous antigens and/or fluorescent proteins. This method had been shown to be particularly beneficial for revealing brain molecular architecture and function (Oberti et al. 2011; Rah and Choi 2022).

### SBF-SEM

As another section-based means of obtaining serial scanning electron micrographs of large volumes, serial block face SEM (SBF-SEM) was introduced in 2004 (Denk and Horstmann 2004); for a review, see Wanner et al. (2015). For this approach, a mini ultramicrotome with a diamond knife is placed inside the chamber of a scanning electron microscope. The ultramicrotome automatically cuts serial sections, and the resulting fresh block faces are imaged while the sections are discarded. Because each section is discarded, this allows for a lower section thickness than in array tomography (e.g., 20–30 nm Helmstaedter 2013; Briggman et al. 2011). This route is faster than focused ion beam SEM (FIB-SEM) (described below), and covers larger volumes at the same time (Zankel et al. 2014), allowing the examination of the neuronal connectivity in blocks of considerable lengths (e.g., ~ 400 µm) (Wernitznig et al. 2022) and volumes of up to 500,000 µm^3^ (Motta et al. 2019).

### FIB-SEM

As reviewed by Kizilyaprak et al. (2019), block faces are also visualized when FIB-SEM is used, but the key difference with respect to ATUMTome SEM is that an instrument with two beams is needed: a focused ion beam (commonly, from gallium) and an electron beam. The FIB repeatedly etches away a specific part of the block, and each new block face is sequentially scanned with the electron beam (Kizilyaprak et al. 2019). The first time a FIB was combined with SEM analysis of biological samples was as early as 1993 (Young et al. 1993), but it took another 13 years until the technology was established for serial sectioning samples in combination with SEM visualization and TEM sample preparation (Heymann et al. 2006), and for 3D reconstruction of large brain volumes (Knott et al. 2008). The advantages over block face SEM are the greatly improved axial resolution (down to as small as 3–5 nm, depending on various parameters), but a disadvantage is that it is significantly more time-consuming.

### Tomography

The potentially highest-resolution 3D imaging technique available is electron tomography, as reviewed for biological imaging by Gan and Jensen (2012) and Marco et al. (2004). For transmission electron tomography, the sections are tilted and images are recorded under different tilt angles, thus projecting the structures inside the sections under different orientations. A computer-based (weighted) back-projection then allows the creation of a 3D model of these structures (Marco et al. 2004), based on virtual parallel slices through the object. A typical transmission electron tomogram is based on 41 up to 141 micrographs taken at tilt angles between –60° to –70° and +60° to +70° (depending on the settings for the tilt increment and range).

Although electron tomography offers the highest resolution of all the 3D techniques described here, TEM tomography also suffers from a few shortcomings. In the *x* and *y* direction, the field of view is rather small, while in the *z* direction, both the beam penetration and focal depth are limited, and the restricted tilt range will result in artifacts in the reconstructed volume (missing wedge/cone). A typical TEM tomogram is thus made with semithin sections limited axially to 200–400 nm. As described below, STEM tomography allows a greater sample thickness than TEM tomography. For STEM tomography, in the current context of thick sections from biological objects, the electron microscope is tuned such that the electron beam forms a “pencil,” i.e., in the form of an almost parallel beam. In STEM, image formation is not achieved by using an imaging lens, the objective lens; hence, there are no spherical aberrations in the images (whether the object is tilted or not). The images are taken by recording the unscattered (bright field) and scattered (dark field) electrons separately on dedicated detectors (or detector areas). These signals are recorded sequentially for each pixel in the relevant sample area. In direct comparison with TEM tomography, sections of biological objects can be as thick as 1000 nm (or even thicker), and highly tilted images are almost “in focus” in all parts.

Regardless of which EM tomography option is chosen, the procedure always includes alignment and reconstruction, the result of which is a stack of slices through the object under investigation, followed by segmentation of the data cube and volumetric analysis (Wacker and Schroeder 2013).

### Solving scientific questions with 3D information from large volumes

In this section, we review in detail how each group solved its respective scientific question.

### Neuroanatomy and cellular structure of the *C. elegans* dauer nervous system investigated with 3D EM techniques (Britz et al. 2021a)

Britz and colleagues examined the architecture of the *C. elegans* nervous system using FIB-SEM. Under harsh conditions, its 2.5-day reproductive cycle changes to the dauer (enduring) condition. Dauer larvae have pronounced developmental nervous system plasticity regarding both morphology and physiology (Britz et al. 2021b).

Following high-pressure freezing–freeze substitution (HPF-FS) (Schieber et al. 2017) and FIB-SEM, both volumetric and skeleton reconstruction of the anterior sensory apparatus were done, and the anterior sensilla region was described in detail on the basis of these 3D reconstructions. In this analysis, Britz et al. focused on the dauer-specific dendritic branching of IL2 neurons and additionally investigated dauer-specific synaptic connectivity. The aim was to analyze the plasticity of dauer-specific connectivity in comparison with other stages. The IMOD software package was used for volumetric segmentation of stacks containing 10,000 images as described by Kremer et al. (1996). Cell tracing, tracing of synapses, and labeling of pre-and postsynapses were done in CATMAID, while TrackEM and Fiji were used to stitch images. Chemical synapses are traditionally detected in electron micrographs on the basis of structural features, viz. presynaptic and postsynaptic densities, presynaptic vesicles in clusters, and synaptic cleft (Burette et al. 2015). However, all three features are not always visible. In their work, Markert et al. (2016) used array tomography in combination with immunolabeling and superresolution microscopy (SIM and dSTORM) to fill the gap and add molecular components to enable structural synapse identification. Innexins (proteins that form electrical synapses in invertebrates such as *C. elegans*) were labeled fluorescently and imaged using structured illumination microscopy (SIM). This was followed by contrasting and carbon coating, imaging by SEM, and finally correlation of data obtained by light and electron microscopy. The same approach in combination with dSTORM was applied to achieve an even more precise localization by correlation. This work contributes to the list of almost fully mapped connectomes of other larval stages of *C. elegans* by adding the missing dauer stage.

### Large-scale electron tomographic reconstruction of mitotic spindles (Botzenhart et al. 2021)

Botzenhart and colleagues study the spindle apparatus and chromosome segregation in *C. elegans* and human cell lines such as Hela, U2OS, or RPE-1. The major goal of this group is to reconstruct the spindles to see how they are organized in different cell lines. For this, it is necessary to distinguish prometaphase from metaphase and metaphase from early anaphase, which are very rare or transient events (Botzenhart et al. 2021). They established an elegant workflow whose crucial and foremost step is to prepare and enrich the samples using a sophisticated protocol (Kiewisz et al. 2021). In brief, the sapphire discs are prepared for HPF and FS by a series of cleaning and coating steps. To enrich the cells, the authors used a shake-off technique that allows the removal of dead cells and cells that already entered mitosis and allows the collection of only rounded cells which are about to enter mitosis. A small 3D-printed chamber with space for four sapphire discs for incubation was generated. This was followed by high-pressure freezing to vitrify the water in the cells and thus prevent ultrastructural damage due to water expansion. Next, during freeze substitution, cells are stained using a cocktail of 1% OsO_4_ and 0.1% uranyl acetate in acetone saturated with a histological dye (methylene blue) to stain DNA. Samples are embedded in epoxy resin, and the cells on the top layer of the resin block are prescreened using a light microscope to locate the cells of interest in desired mitotic stages. The next step in this workflow is preparing the data for tomography. As a first step, suitable cells stained by methylene blue are identified via optical sectioning by a confocal light microscope. Once the area of interest is marked, serial sections with a thickness of 300 nm are obtained by ultramicrotomy. They imaged the stack of serial sections in TEM and correlated the electron micrographs with fluorescent signals to confirm that no sections were missed out (CLEM), before applying electron tomography. Dual-axis electron tomography was used to reduce the missing cone problem. First a series of images of serial sections containing the cells of interest from +65° to −65° in steps of 1° were imaged using TEM operated at 300 kV. The sample was then rotated by 90° and a second tomogram recorded, to obtain two stacks of images with two different rotations. For 3D reconstruction, the “Batch tomography” option of the IMOD software, which allows serial processing of several regions of interest, was used, thus widening the volume under investigation. The “flatten volume” setting in this software was used to flatten the tomogram and label the border. Since the goal is to reconstruct the mitotic spindles, they used the ZIB Amira software package that enables semiautomatic microtubule segmentation to reconstruct the microtubules (Lantzsch et al. 2021). Using this approach, the time taken for tomogram acquisition, reconstruction, and microtubule tracing was significantly reduced, by 60% (Botzenhart et al. 2021). Lastly, they resolved the problem in question by using an open-source online platform called ASGA (Automatic Spatial Graph Analysis) to analyze their tomographic data. ASGA could be used to analyze several characteristics of, in this case, microtubules, including their distribution, number, microtubule interaction, and inter-kinetochore distance.

### Multimodal analysis of intercellular interactions to reveal microglial contactomics in three dimensions (Cserép et al. 2021)

Cserép and colleagues used electron, light, and superresolution microscopy to investigate spatial microglial connectomics. They identified an interaction site between neuronal cell bodies and microglial processes and connected the activity of neuronal mitochondria with microglial junction formation (Cserép et al. 2021, 2020).

In vivo imaging of mouse neocortical neurons using two-photon (2P) microscopy revealed long-lasting microglial interaction sites at the neuronal soma but rarely at dendritic protrusions or synapses. Further experiments using multiple microscopy techniques (STORM, immunogold localization of targets by TEM, etc.) showed that neuronal potassium channels Kv2.1 and Kv2.2 clusters, known to anchor intracellular organelles to the plasma membrane (Kirmiz et al. 2018), as well as the microglial purinoceptor (P2Y12), are highly involved in the tight neuronal and microglial interaction. Surprisingly, these unique morphofunctional junctions contain accumulations of mitochondria and mitochondria-associated vesicles that are anchored by electron-dense regions to the contact sites.

In particular, P2Y12 seems to be involved in the tight connection between neuronal soma and microglia, because a clear correlation between P2Y12 clusters and the distance between neuron and microglia membranes was found by 3D analysis of tomograms of P2Y12 immunogold-labeled samples. Measurements of nicotinamide adenine dinucleotide (NADH) autofluorescent levels showed that these somatic junctions were responsible for spatially increasing mitochondrial activity in neurons depending specifically on the expression of P2Y12. Confocal laser scanning microscopy (CLSM) was used to visualize that vesicular nucleotide transporter, known to be responsible for somatic vesicular adenosine triphosphate (ATP) release in neurons, was localized to the mitochondrial and the microglia-contacted neuronal membrane.

Inhibition of P2Y12 by PSB0739 yielded reduced neuronal somatic junctions to microglia, and the P2Y12-dependent recruitment of microglia (dependent on neuronal activity) was shown using an in vivo 2P microscopy designer receptor exclusively activated by designer drug (DREADD) approach. These results show the important crosstalk of neurons and microglia, to preserve and react to metabolic and acute energy demands. Acute brain injury after stroke resulted in P2Y12-dependent microglial protection of neurons by declustering of Kv2.1 channels and higher coverage of neuronal soma with microglial contact sides. Inhibition of P2Y12 by PSB0739 also increased neuronal Ca^2+^ levels measured with the intensiometric genetic encoded Ca^2+^ indicator GCaMP6f and increased lesion volume after brain injury.

### Development of a 3D liquid-phase STEM method to study cell–biomaterial interaction (Bekel et al. 2021)

Bekel and colleagues studied the interactions between cells and biomaterials and aimed to reduce the changes in cells during sample preparation using the liquid-phase SEM technique. For this, 3T3 cells were cultured on a gold TEM grid coated with a fibronectin–carbon film. Following this, the cells were fixed in 4% glutaraldehyde for 30 min and stored at 4 °C until image acquisition. Bekel et al. designed a device to acquire images in tilt series for tomographic reconstructions (Jiao et al. 2019; Masenelli-Varlot et al. 2014). Bekel et al. proceeded to record images by environmental scanning electron microscopy (ESEM) at 15 kV, maintaining humidity at or above 70% and a temperature of 4 °C, so that the cells did not dry out. Bright-field and high-angle annular dark-field images were recorded at different tilt angles. They used ImageJ to align the tomograms and the TomoJ plugin of ImageJ to compute volumes of the tomograms. Data segmentation was done in ImageJ and 3D Slicer. The advantage of this method is that it enables 3D image acquisition of liquid samples. Since no staining or embedding steps are involved, this approach greatly cuts the time and lengthy protocols required for pre-embedding and embedding steps. Bekel and his team showed that their approach for electron tomography using liquid-phase ESEM enables 3D analysis of whole, intact, hydrated eukaryotic cells, including their overall morphology (Bekel et al. 2021).

### Three-dimensional investigations on ciliogenesis in hRPE cells (Rachel et al. 2021a), and the shape and structure of Archaeal cells, analyzed by STEM tomography (Rachel et al. 2021b)

Rachel et al. primarily used human retinal pigment epithelial (hRPE) cells to meet their first goal. The group focuses on investigating the recruitment of a key protein in hRPE cells, the small GTPase RAB8A, to the cilium. Rachel et al. opted for CLEM to achieve their aim. First, in adherently grown cells (fixed using formaldehyde) the position of the fluorescently labeled RAB8A protein relative to developing cilia was analyzed using light microscopy. Subsequently, the cells were fixed by adding glutaraldehyde, postfixed and stained with OsO_4_ and uranyl acetate, and embedded in resin. Serial sections were used to locate the protein in its subcellular context by STEM tomography. STEM tomograms of sections with a thickness of 600 nm (or even thicker) were generated and analyzed using the eC-CLEM software package. The advantage of combining STEM with CLEM is that STEM allows tomogram acquisition of sections with thickness of up to 900 nm (or even 1000 nm) at 200 kV. Hence, they could visualize the localization of RAB8A at the cilium (Buerger et al. 2021). The second goal of the group was to visualize subcellular compartments and cell appendages in prokaryotes at the ultrastructural level. *Pyrococcus furious*, an archaeon expressing numerous archaella, was cryo-immobilized by HPF to preserve the natural shape and ultrastructure, followed by FS for best ultrastructural preservation, and embedding in resin. Sections up to a thickness of 900 nm were collected via ultramicrotomy, and STEM tomograms were generated using dual-axis tilting. This enables an increase in the focal depth and the analysis of large volumes close to the size of a complete cell (Rachel et al. 2020). Rachel et al. could successfully trace the cellular appendages in *P. furious* in an undisturbed state at a resolution of 5 nm, sufficient to resolve individual archaella.

### Large-volume 3D EDS mapping of *Paramecium* (Yamaguchi et al. 2021)

Yamaguchi and colleagues used AT in combination with energy-dispersive X-ray spectroscopy (EDS) to reveal the 3D structure of *Paramecium* and 3D EDS maps of its phagosome with gold, silver, and silica nanoparticles.

*Paramecium* was first cultivated with gold, silver, and silica nanoparticles, for 1 h each (size of 40, 40, and 50 nm, respectively). Following fixation and contrasting (glutaraldehyde, OsO_4_, and uranyl acetate), the samples were embedded in resin. Sections of 200 nm (283 slices in total) were mounted on ultraflat carbon substrate, and the contrast enhanced with lead citrate and uranyl acetate. Using a BSE detector, serial two-dimensional images were acquired by field-emission scanning electron microscopy (FE-SEM; incident voltage 7 keV), and 3D elemental mapping was performed by EDS mounted inside the FE-SEM. By stacking the 2D images, 3D volumes of whole *Paramecium* cells and in-parallel 3D elemental maps were obtained with so far unparalleled data quality.

Yamaguchi and colleagues were successful in obtaining 3D data of *Paramecium* cells in a volume of 226 × 90 × 56 µm^3^, including several phagosomes. The 3D elemental map of one of the phagosomes revealed the distribution and amount of gold and silica nanoparticles, while silver particles were not detected. The difference in the amount of the mentioned metal nanoparticles could be due to selective ingestion by *Paramecium* or the order of adaptation for each element.

## Discussion

**Fig. 1:**
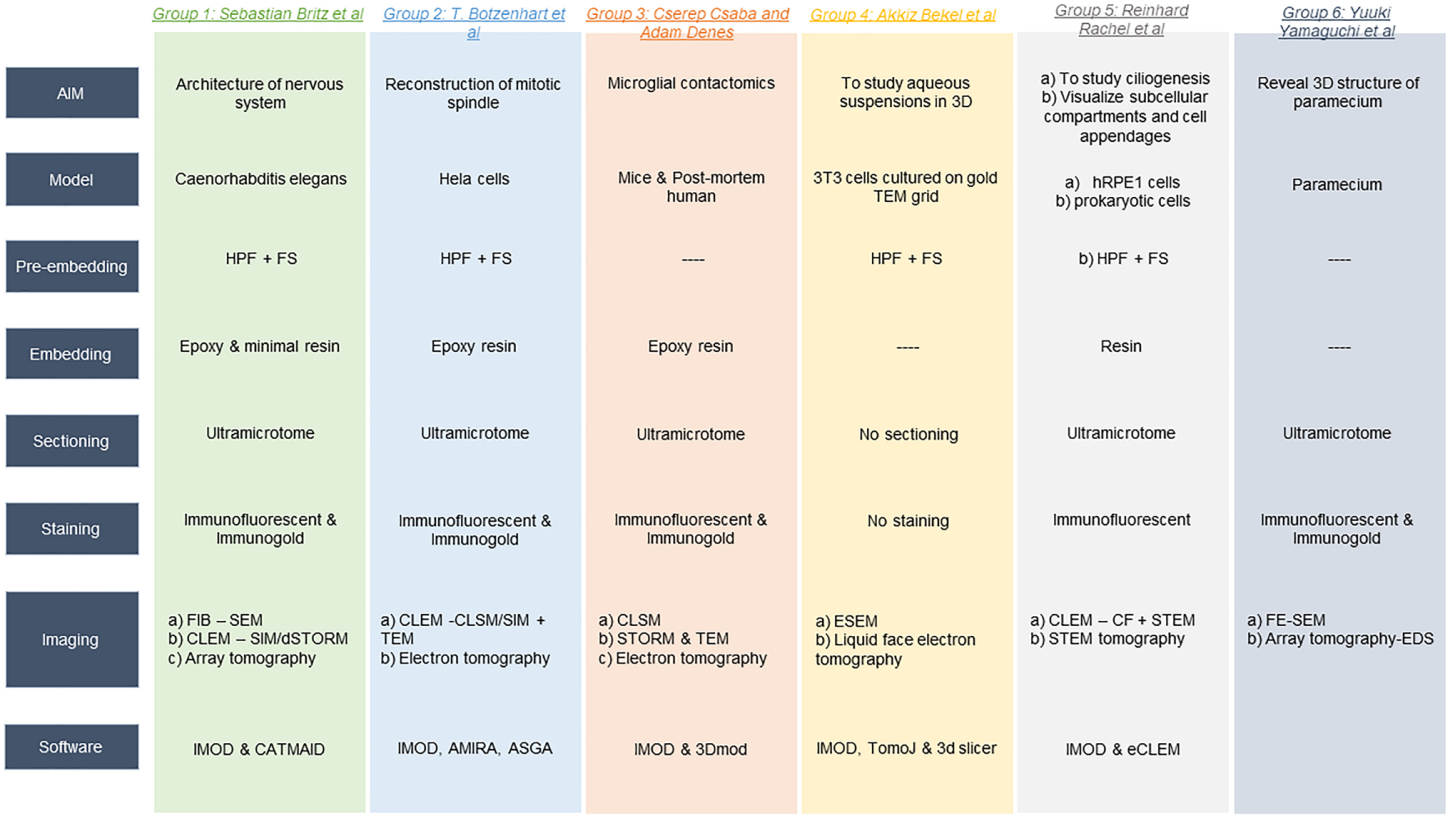
Overview of the workflow of each group

Increasing the volume and the information content of high-resolution 3D visualizations is a difficult task. With the few selected examples listed in this review, we show that the solutions vary and each laboratory has chosen its own approach to overcome this hurdle. The method chosen depends on the scientific questions, the biological system, and the equipment available. Thus, the scientific work summarized herein was obtained by using various microscopy techniques. In all cases, more than one microscopy technique was used to answer the respective scientific questions (Fig. 1); i.e., combining several different microscopy techniques was the key to success. For example, structural information obtained by electron microscopy was complemented with molecular localizations obtained by fluorescence microscopy (Rachel et al. 2021a; Markert et al. 2016). This offered a way to overcome the limitations of each single method. Several scientific questions were solved by 3D reconstructions based on SEM micrographs of multiple, serial sections. Image stacks were obtained by FIB-SEM (Britz et al. 2021a) or array tomography (Yamaguchi et al. 2021). Both techniques provide the opportunity to reconstruct almost unlimited axial lengths. The number of sections is only limited by the size of the block and the time taken to produce them. In contrast to FIB-SEM and array tomography, TEM tomography has the advantage of a potentially higher resolution, although the volume under investigation is limited by the section thickness. This issue was resolved, for example, in the work by Botzenhart et al. (2021) by modifying TEM tomography to analyze unusually large cell volumes. Tomograms of each section were acquired as individual tiles, 3D reconstructed, and later stitched together into a larger, single tomogram to expand the volume in the *x*/*y* direction and by stacking the acquired tomograms of serial sections in the *z* direction to fully reconstruct the recorded cellular object (Lindow et al. 2021). In another example, the issue of limited section thickness was addressed by using a different route: on samples which had first been investigated by fluorescent light microscopy, the 3D analysis was done by analyzing up to 900-nm-thick sections using STEM tomography (Buerger et al. 2021).

While significant improvements have been made in terms of sample volume size, significant scientific progress was achieved by creatively combining different types of electron and fluorescent microscopy. CLEM was used by Britz and colleagues in their previous work (Markert et al. 2016) to improve synapse identification, and Botzenhart et al. (2021) used it to obtain 3D reconstructions of the spindle apparatus. Cserép et al. (2020) utilized 2P microscopy to perform in vivo imaging of mouse neocortical neurons. CLSM and STORM were applied to visualize the subcellular distribution of various proteins at microglia–neuron contact sites (Cserép et al. 2021). Bekel et al. (2021) used ESEM to visualize cells that were only chemically fixed to be imaged in ESEM, circumventing time-consuming dehydration and embedding protocols. Yamaguchi et al. (2021) utilized FE-SEM and AT-EDS to locate/visualize gold and silica nanoparticles in 3D-reconstructed *Paramecium*.

In cases when the analysis was planned to be done with fluorescently labeled biomolecules, the same sample was investigated by both (one variation of) fluorescent light and (one variation of) electron microscopy, i.e., by CLEM. CLEM is a time-consuming technology, in particular as the workflow for sample preparation must be adapted to make it compatible with and optimized for both microscopy techniques. However, with technical improvements, CLEM becomes the method of choice when both structural and functional components of a biological sample are needed to answer a particular question. When integrated into one system, the combination of fluorescent and electron microscopy offers a more convenient option to perform CLEM, and commercial solutions are already available and constantly improving.

## Abbreviations

2P: Two photon
3D: Three-dimensional
ASGA: Automatic spatial graph analysis
AT: Array tomography
ATP: Adenosine triphosphate
ATUMTome: Automated tape collection ultramicrotome
BSE: Backscattered electrons
Ca^2+^: Calcium
CAT: Correlative array tomography
CLEM: Correlative light and electron microscopy
CLSM: Confocal laser scanning microscope
DNA: Deoxyribonucleic acid
DREADD: Designer receptors exclusively activated by designer drug
dSTORM: Direct stochastic optical reconstruction microscopy
EDS: Energy-dispersive X-ray spectroscopy
EM: Electron microscopy
ESEM: Environmental scanning electron microscopy
FE-SEM: Field-emission scanning electron microscopy
FIB: Focused ion beam
FS: Freeze substitution
GTP: Guanosine triphosphate
HPF: High-pressure freezing
hRPE: Human retinal pigment epithelial
IL: Interleukin
NADH: Nicotinamide adenine dinucleotide hydrogen
OsO_4_: Osmium tetroxide
P2Y12: Purinergic receptor P2Y12
PSB0739: Antagonist of P2Y12
RPE-1: Retinal pigment epithelial-1
SBF: Serial block face
SEM: Scanning electron microscopy
SIM: Structured illumination microscopy
STEM: Scanning transmission electron microscopy
STORM: Stochastic optical reconstruction microscopy
TEM: Transmission electron microscopy
U2OS: Human bone osteosarcoma epithelial cells
